# Stroke-Related Changes in the Complexity of Muscle Activation during Obstacle Crossing Using Fuzzy Approximate Entropy Analysis

**DOI:** 10.3389/fneur.2018.00131

**Published:** 2018-03-12

**Authors:** Ying Chen, Huijing Hu, Chenming Ma, Yinwei Zhan, Na Chen, Le Li, Rong Song

**Affiliations:** ^1^Key Laboratory of Sensing Technology and Biomedical Instrument of Guang Dong Province, School of Engineering, Sun Yat-sen University, Guangzhou, China; ^2^Department of Rehabilitation Medicine, Guangdong Engineering Technology Research Center for Rehabilitation Medicine and Clinical Translation, The First Affiliated Hospital, Sun Yat-sen University, Guangzhou, China; ^3^Guangdong Work Injury Rehabilitation Center, Guangzhou, China; ^4^School of Computers, Guangdong University of Technology, Guangzhou, China

**Keywords:** fuzzy approximate entropy, obstacle crossing, stroke, gait, electromyography

## Abstract

This study investigated the complexity of the electromyography (EMG) of lower limb muscles when performing obstacle crossing tasks at different heights in poststroke subjects versus healthy controls. Five poststroke subjects and eight healthy controls were recruited to perform different obstacle crossing tasks at various heights (randomly set at 10, 20, and 30% of the leg’s length). EMG signals were recorded from bilateral biceps femoris (BF), rectus femoris (RF), medial gastrocnemius, and tibialis anterior during obstacle crossing task. The fuzzy approximate entropy (fApEn) approach was used to analyze the complexity of the EMG signals. The fApEn values were significantly smaller in the RF of the trailing limb during the swing phase in poststroke subjects than healthy controls (*p* < 0.05), which may be an indication of smaller number and less frequent firing rates of the motor units. However, during the swing phase, there were non-significant increases in the fApEn values of BF and RF in the trailing limb of the stroke group compared with those of healthy controls, resulting in a coping strategy when facing challenging tasks. The fApEn values that increased with height were found in the BF of the leading limb during the stance phase and in the RF of the trailing limb during the swing phase (*p* < 0.05). The reason for this may have been a larger muscle activation associated with the increase in obstacle height. This study demonstrated a suitable and non-invasive method to evaluate muscle function after a stroke.

## Introduction

Stroke, a leading cause of disability, often leads to functional limitations in the activity of daily living (ADL). Stroke survivors have a high risk of falling during all poststroke stages (1). Mackintosh et al. found that 36% of community-dwelling elderly people with chronic poststroke symptoms reported falling in the past year, which is significantly more than 24% of the healthy controls (2). Rehabilitation intervention offers beneficial effects on motor recovery after a stroke (3) and can reduce the risk of falling (4). A better understanding of motor function impairment in stroke survivors will help design effective recovery strategies during rehabilitation to reduce the incidence of falling.

Obstacle crossing is a complex walking ADL and requires sufficient foot obstacle clearance for the swinging limb, stability of the stance limb (5), and coordination of the whole body to prevent the loss of balance. Half of all stroke subjects either lose their balance or make casual foot contact with the obstacle during crossing (6), which indicates that obstacle crossing threatens the safety of patients after a stroke. Many studies based on the kinematic indices have been conducted to analyze stroke patients’ gaits. Kerrigan et al. examined the joint angles of stroke patients during level walking to quantitatively define the most commonly used strategy termed circumduction (7). Lu et al. investigated the motor performance in high-functioning poststroke patients during obstacle crossing and found that stroke survivors appeared to adopt a specific symmetric kinematic strategy with an increased pelvic posterior tilt and swing hip abduction (8). Said et al. quantified the modifications of kinematic characteristics in stroke survivors during obstacle crossing and found that stroke survivors had reduced toe-obstacle clearance and closer horizontal distance after clearance with increased crossing time compared to healthy controls (9).

Previous studies based on the kinematic analysis identified a significant number of stroke-related features for obstacle crossing. Further information about muscle function requires electromyography (EMG) signals, which can be recorded from the muscle surface (10). EMG analysis based on time and frequency domains was widely used in previous studies. Zhai et al. proposed a self-recalibrating classifier of hand movements based on the convolutional neural network using short latency dimension-reduced sEMG as an input (11). Chen and Yang successfully reconstructed drawings of trace reconstructions using a novel three-step hybrid model based on the root mean square (RMS) of seven-channel EMG signals and a gene expression program (12). Kisielsajewicz et al. found that the coherence between synergist muscles in the affected upper limb of stroke patients was lower than that of healthy subjects during reaching tasks (13). Our previous results showed greater muscle activation levels, increased muscle co-contraction, and lower mean power frequencies in persons after a stroke compared to controls during obstacle crossing. These findings indicated that abnormal muscle activation patterns might contribute to difficulties in maintaining balance during obstacle crossing (14). Since the generation of EMG signals is non-linear (15), simple linear modeled features, such as the RMS, integrated EMG, and mean power frequency, reported recently are limited in characterizing muscle dynamics (16). Some non-linear methods have been introduced to analyze the EMG signals, including fractal dimension, average maximum finite-time Lyapunov exponents, and recurrence quantification analysis (15, 17, 18). However, these non-linear dynamic methods usually require very large data sets to achieve reliable results. This may lead to spurious results when applied to small data sets from experiments (19). To solve this problem, entropy-based methods, such as approximate entropy (ApEn), sample entropy (SampEn), and fuzzy approximate entropy (fApEn), have been introduced to analyze EMG signals (19–21). For example, Zhang et al. used SampEn to detect the onset of muscle activity and found that it was more robust than the RMS method (21, 22). It was further used to examine the EMG-torque relation in the complexity domain. This demonstrated that complexity analysis is a novel tool to examine neuromuscular changes after stroke (23).

Entropy was first introduced by Shannon and later termed information entropy (24). Kolmogorov then developed K-S entropy based on the information entropy, which was applicable for examining the complexity of systems (25). However, K-S entropy is not useful for the analysis of measured signals because these signals are noise, and K-S entropy is unable to analyze noisy signal (26). Pincus subsequently introduced ApEn, which is applicable to noisy and small data sets (26). Although ApEn has many advantages compared with linear analysis methods, it is biased. To solve this problem, SampEn was then developed based on ApEn (27). SampEn is less dependent on the size of data sets and shows better relative consistency, but SampEn(*m, r, N*) is not defined in the case of small *N* and *r* (27). Chen et al. later developed fApEn as another complexity analysis method. It combines Zadeh’s fuzzy sets with entropy-based methods (19). Due to its excellent robustness and consistency (28), fApEn can analyze muscle function in patients with neuromuscular disorders. Ao et al. found that the fApEn values in the elbow muscles were lower compared to healthy controls (29). Sun et al. found that fApEn values increased with force-generating capacity in stroke survivors during robot-aided rehabilitation training sessions (30). To date, there are limited studies on the dynamics of muscle function during complex tasks, such as obstacle crossing following stroke. This is critical to daily living.

In this study, fApEn was used to analyze the EMG signals recorded from eight muscles of the lower limb of poststroke subjects and compared those with healthy subjects when performing obstacle crossings tasks at different heights. This study aimed to investigate the alterations in the complexity of the EMG signals between the two groups and between different heights during the task. It also aimed to identify dynamic muscle function changes after stroke. Our hypothesis was that the complexity of the generated EMG signals would decrease due to muscle damage after stroke. The complexity would increase along with the obstacle height due to the underlying mechanisms of muscle activation. The correlation between the fApEn values of the EMG signals and the clinical scales could provide further details regarding muscle function after stroke.

## Materials and Methods

### Participants

Five poststroke subjects with at least 3 months onset prior to data collection and were capable of stepping across a 30% leg length height obstacle were recruited. In addition, eight healthy subjects of similar heights and gender participated in the experiment as controls. The Fugl-Meyer Assessment (FMA) and Berg Balance Scale for lower extremities were used to evaluate the motor function of the poststroke subjects. The clinical scales assessments were conducted by an experienced physiotherapist. The basic information for the poststroke patients is shown in Table 1. This study was approved by the Ethics Committee of the First Affiliated Hospital of Sun Yat-sen University. This study was conducted in accordance to the Declaration of Helsinki. All subjects provided written informed consent prior to enrollment.

**Table 1:** Background data of the stroke survivors.

Subject	Age (years)	Duration (months)	Paretic hemisphere	Clinical scales	
				FMA-LE	BBS
1	45–50	26	L	28	47
2	45–50	4	L	26	43
3	40–45	3	R	18	33
4	70–75	3	R	27	41
5	50–55	3	R	22	41

*To avoid indirectly identifiable patient data, the genders of stroke group were presented as four males and one female; and the ages were presented as a range*.

*BBS, Berg Balance Scale; FMA, Fugl-Meyer assessment scale of the motor function in paretic low-extremity; L, left; R, right*.

### Apparatus

**Figure 1:**
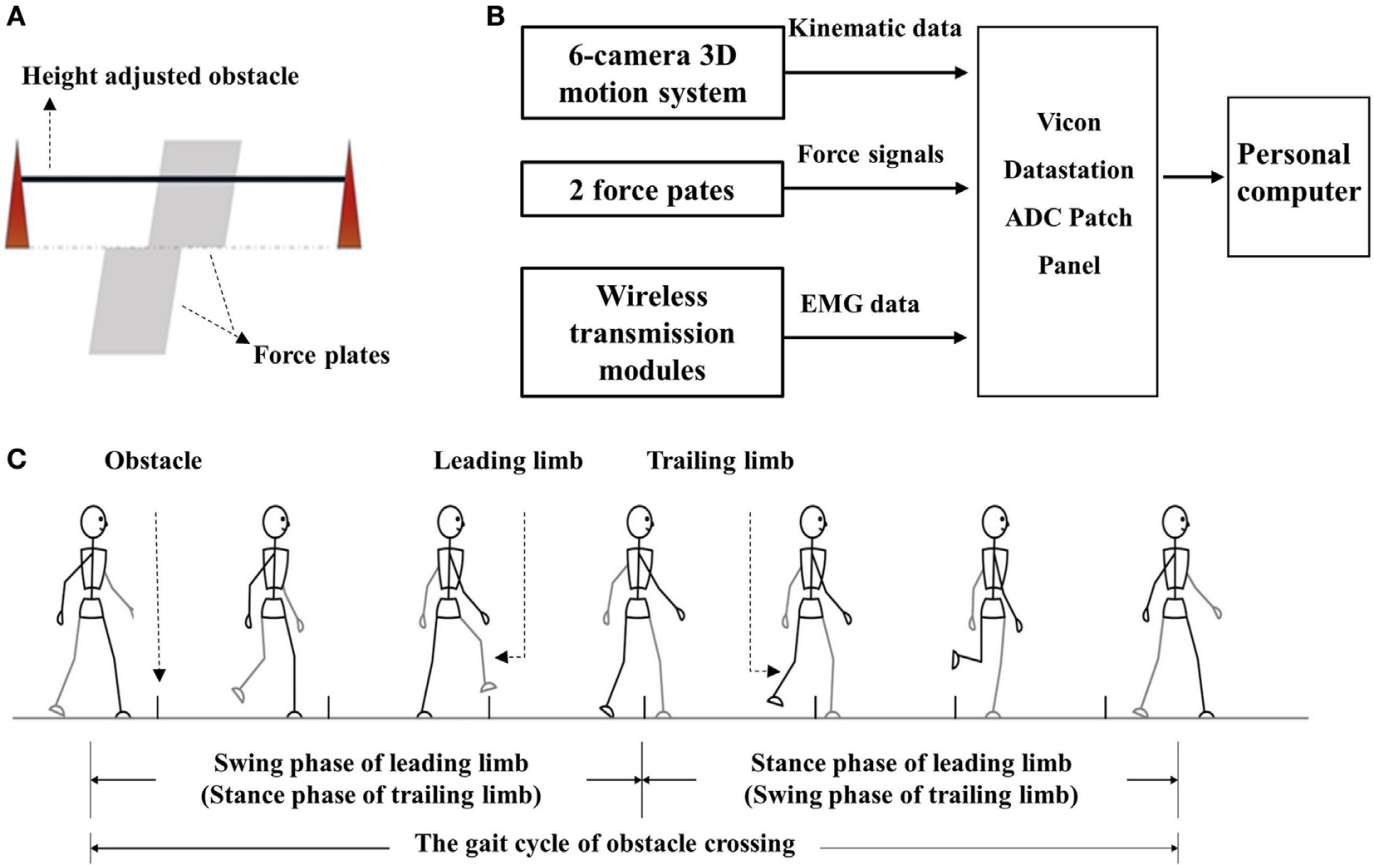
**(A)** The diagram of obstacle and force plate; **(B)** flow diagram of the procedure of data collection and storage; and **(C)** diagram of the gait cycle of obstacle cycle.

The kinematic data were recorded by a 6-camera 3D motion analysis system (Vicon Motion Systems, Oxford, UK). Two force plates (AMTI, Watertown, MA, USA) situated in the middle of the path were used to record the force signals. The height-adjustable obstacle was placed between them. A diagram of the two force paths and obstacles is presented in Figure 1A. EMG data were recorded from the rectus femoris (RF), biceps femoris (BF), tibialis anterior (TA), and medial gastrocnemius of both sides for all subjects using preamplified wireless transmission modules. Figure 1B shows the flow diagram of the procedure for data collection. The apparatuses used in this study were the same as our previous experiment. A detailed description was provided in our previous study (14). The sample frequency for Vicon cameras was 100 Hz and 1 kHz for the force plates and EMG modules.

### Procedure

The subjects’ heights, body weights, and leg lengths were first measured and recorded before kinematic data collection. The distance from the anterior superior iliac spine to the lateral malleolus was measured as leg length. This was then used to calibrate the height of the obstacle of each individual. Thirty-five 15-mm light-reflective markers and silver-silver chloride (Ag-AgCl) electrodes were attached to corresponding positions on each of the subjects. The target skin area was shaved and cleaned with alcohol to obtain better signals before the attachment of the electrodes (14).

The gait trials began after the preparation. The subjects were asked to walk along a volunteered walkway (8 m) at a volunteered speed with bare feet with an obstacle placed at a midway distance. Details of the trials were described previously (14). Figure 1C presents the gait cycle during obstacle crossing. Trials in which the subjects touched the obstacles or asked for assistance were ignored, and three successful trials for each height were recorded.

All subjects completed the maximum voluntary contraction tasks and three different height obstacle crossing tasks. No incident of fall was observed during all trials. The trials where help was received from therapist or the obstacle was touched were discarded. Discomfort or feelings of fatigue were not reported by any subjects during the tasks.

### Data Processing

A 20-Hz low-pass fourth-order Butterworth filter was employed to filter the kinematic and kinetic data. When the toe marker was 2 mm off the ground, this was regarded as the toe-off time. The heel strike time could be recognized according to the change of force signal received by the force platforms. The gait cycle was then divided into two phases or a single lower limb: swing phase and stance phase. The raw EMG signals were collected at a frequency of 1 kHz and then were filtered through a fourth-order Butterworth filter with a frequency band from 10 to 350 Hz. As the frequency of mains of power supply was 50 Hz, a digital notch filter was used to subtract the disturbance of strong electromagnetic fields of 50 Hz that were present in the experiment conducted area.

The fApEn of an *N* sample series is computed as follows:

First, for a given *m*, we formed *m*-dimensional vector sequences:

$$\begin{array}{l} X_{i}^{m}=\left\{\mu \left(i\right),\ldots ,\mu \left(i+m-1\right)\right\}-1/m\overset{m-1}{\underset{j=0}{\sum }}\;\mu \left(i+j\right),\\ \text{where }\mu_{0}\left(i\right)=\frac{1}{m}\overset{m-1}{\underset{j=0}{\sum }}\;\mu \left(i+j\right). \end{array}$$

For every $X_{i}^{m}$, the distance $d_{ij}^{m}$ between the two vectors $X_{i}^{m}$ and $X_{j}^{m}$ is:

(1) $$d_{ij}^{m}={\text{max}}_{k\in \left(0,m-1\right)}\left|\mu \left(i+k\right)-\mu_{0}\left(j\right)-\mu \left(j+k\right)-\frac{1}{m}\overset{m-1}{\underset{j=0}{\sum \;}}\mu \left(i+j\right)\right|.$$

The definition of similarity degree between two vectors $X_{i}^{m}$ and $X_{j}^{m}$ is as follows:

(2) $$D_{ij}=\text{exp}\left(-{\left(\frac{d_{ij}{\;}^{m}}{r}\right)}^{n}\right),$$

where *r* and *n* in Eq. 2 determine the width and gradient of the boundary.

The function φ*^m^* averages the similarity and is defined as follows:

(3) $$\varphi^{m}\left(n,r\right)=\frac{1}{N-m+1}\overset{N-m+1}{\underset{i=1}{\sum }}\text{ln}\left(\frac{1}{N-m+1}\overset{N-m+1}{\underset{i=1}{\sum }}D_{ij}{\;}^{m}\right).$$

The fApEn is then calculated as follows:

(4) $$\text{fApEn}\left(m,n,r,N\right)=\text{ln}\varphi^{m}\left(n,r\right)-\varphi^{m+1}\left(n,r\right).$$

Here, *m* = 2 and *r* = 0.15 ^* ^SD(signal) were set according to the previous study (30).

### Signal Processing and Statistical Analysis

The fApEn values for all the muscles were averaged over three replicates for each subject during each height obstacle crossing. The SD values were also calculated. A two-way (group: control and poststroke × obstacle height: 10, 20, and 30% of leg length) repeated measure of variance (ANOVA) was performed on the fApEn values. A Bonferroni *post hoc* test was used to analyze the fApEn values. Kolmogorov–Smirnov test was applied to the variables. Pearson’s correlation coefficient was used to examine the relationship between the clinical scales and fApEn values when the variables were normally distributed. Spearman’s correlation coefficient was used when the variables were non-normally distributed. The significance level was set at 0.05. All the data were analyzed in SPSS 19.0 statistical software (SPSS Inc., USA).

## Results

**Figure 2:**
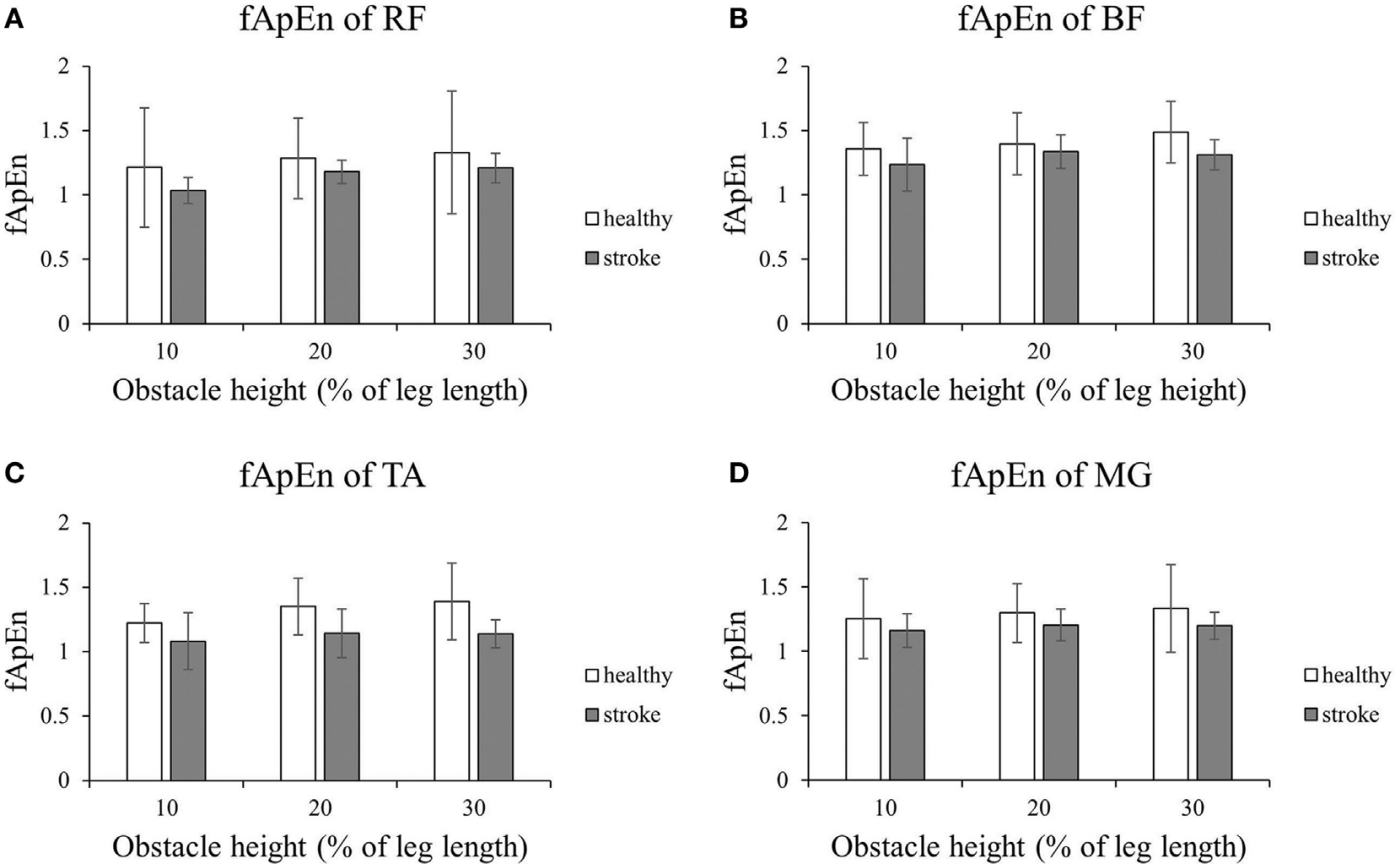
The details of fuzzy approximate entropy (fApEn) values of each height for trailing limb during stance phases. **(A)** The fApEn values of rectus femoris (RF); **(B)** the fApEn values of biceps femoris (BF). **(C)** The fApEn values of tibialis anterior (TA); **(D)** the fApEn values of medial gastrocnemius (MG).

**Figure 3:**
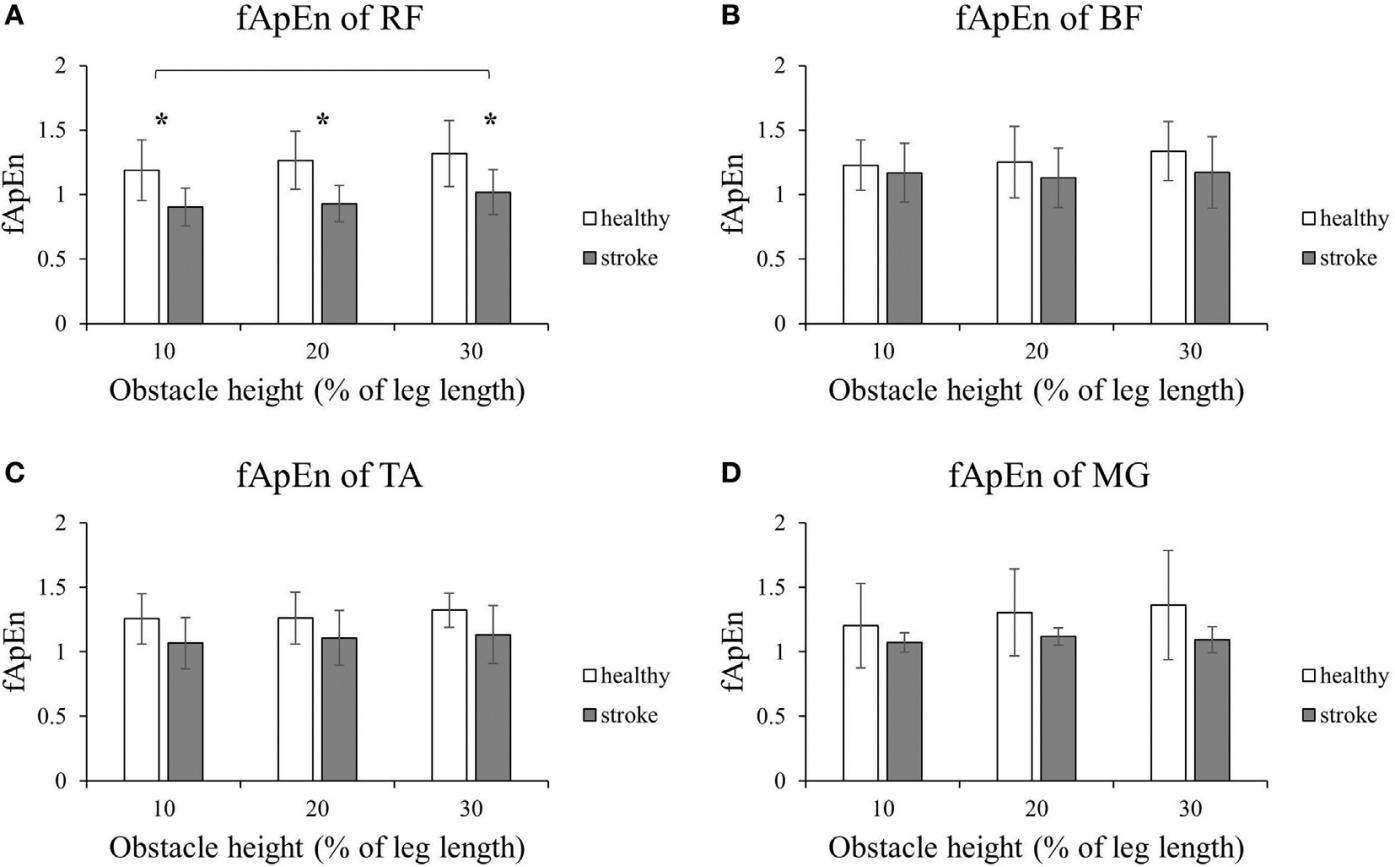
The details of fuzzy approximate entropy (fApEn) values of each height for trailing limb during swing phases. **(A)** The fApEn values of rectus femoris (RF); **(B)** the fApEn values of biceps femoris (BF). **(C)** The fApEn values of tibialis anterior (TA); **(D)** the fApEn values of medial gastrocnemius (MG). *Significant effect between groups. The bar (-) indicates significant effect between heights.

Figure 2 shows that the fApEn values of the four lower limb muscles of the trailing limb during the stance phase. Figure 3 presents the fApEn values of these four muscles during the swing phase. As presented, during this gait cycle, the fApEn values of poststroke subjects were lower than those of healthy controls. In addition, significantly lower fApEn values were found in the RF of poststroke subjects during the swing phase when compared with healthy controls (*p* < 0.05). As shown in Figures 2 and 3, most fApEn values of the four lower limb muscles of the trailing limb increased with the height of the obstacle. Furthermore, a significant increase was observed in the BF during the swing phase when the height of the obstacle increased from 10 to 30% of leg length (*p* < 0.05).

**Figure 4:**
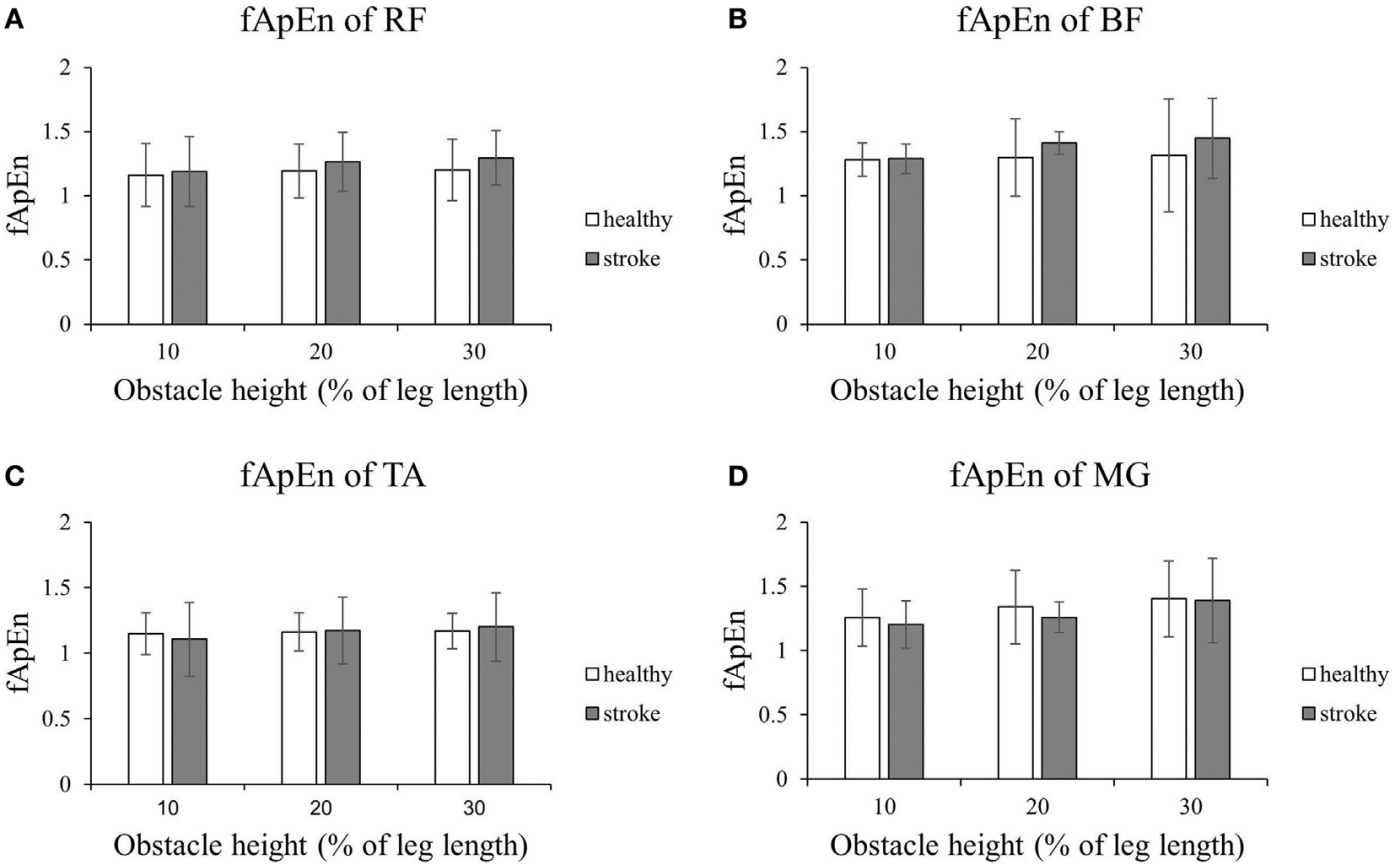
The details of fuzzy approximate entropy (fApEn) values of each height for leading limb during swing phases. **(A)** The fApEn values of rectus femoris (RF); **(B)** the fApEn values of biceps femoris (BF). **(C)** The fApEn values of tibialis anterior (TA); **(D)** the fApEn values of medial gastrocnemius (MG).

**Figure 5:**
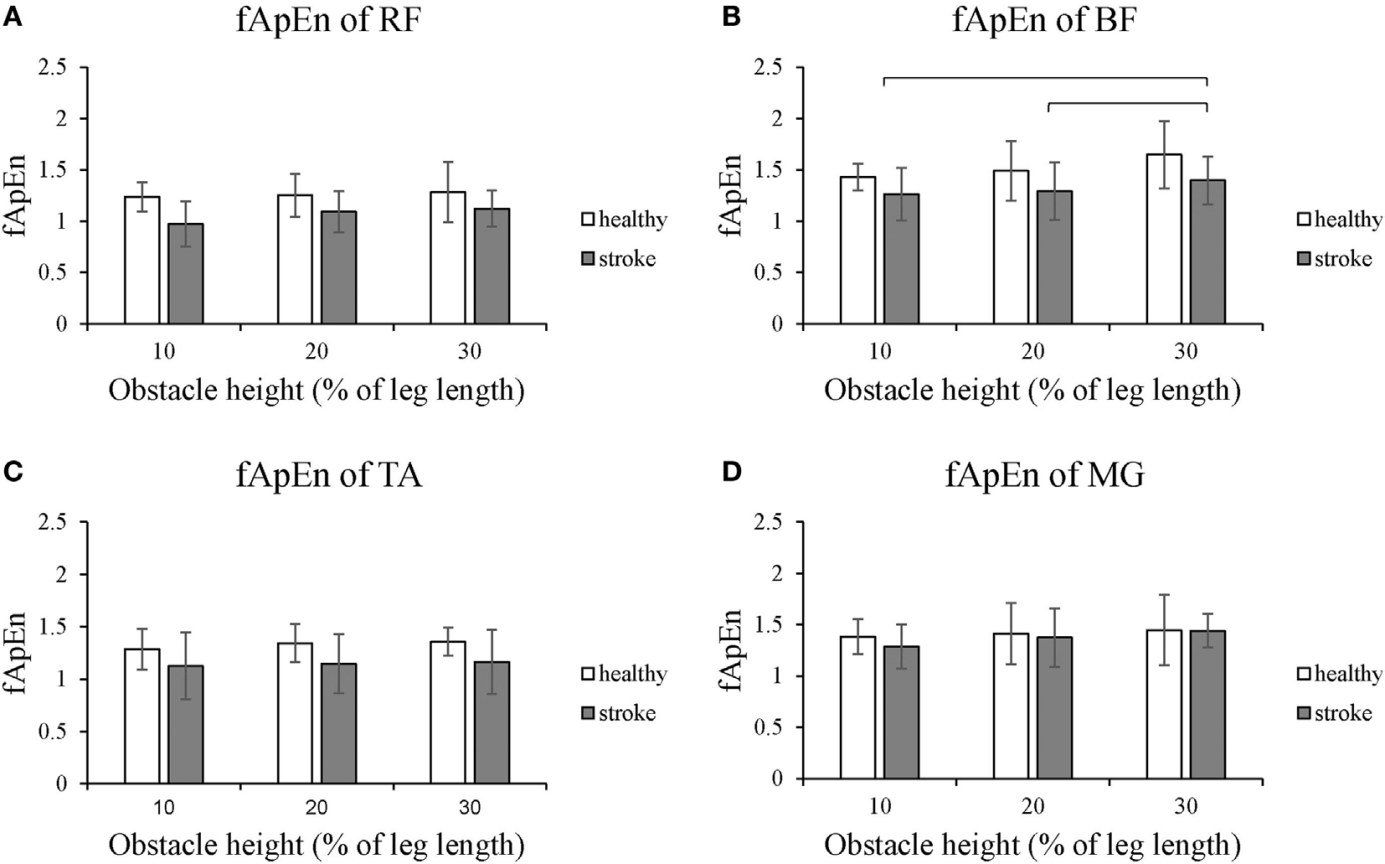
The details of fuzzy approximate entropy (fApEn) values of each height for leading limb during stance phases. **(A)** The fApEn values of rectus femoris (RF); **(B)** the fApEn values of biceps femoris (BF). **(C)** The fApEn values of tibialis anterior (TA); **(D)** the fApEn values of medial gastrocnemius (MG). The bar (-) indicates significant effect between heights.

Figures 4 and 5 present the results of the four lower limb muscles of the leading limb during the swing phase and stance phase. As shown in Figure 4, during the swing phase, the fApEn values of the BF and RF in poststroke subjects were higher than in the healthy controls, and this result was similar for the TA during the swing phase when the obstacle height was 20 and 30% of leg length. Meanwhile, during the swing phase, fApEn values for all four muscles were lower in poststroke subjects compared with healthy controls. However, these differences between groups were non-significant (*p* > 0.05). Similar to the results of the trailing limb, the increase in the fApEn of the muscles with the obstacle height was also found in the leading limb. In addition, as presented in Figure 5, the fApEn value of the BF during the stance phase was statistically significantly greater when the obstacle height was 30% of leg length compared with 10 and 20% (*p* < 0.05). The correlations between fApEn and the two clinical scales were not statistically significant.

## Discussion

We recorded EMG signals and calculated the fApEn values of four lower limb muscles of poststroke subjects during different phases of obstacle crossing at different heights. Complexity change in muscle activations were then compared between poststroke subjects and healthy controls when they conducted this challenging task.

### fApEn Values Change after a Stroke

The decreased fApEn values of EMG signals in poststroke subjects could be explained by that muscles were damaged, and they became disused after stroke. This might lead to their degenerated internal structure (31). The motor unit properties changed because of the reduced corticofugal output from the paretic hemisphere (32). In addition, the number of functioning motor units and the firing rate decreased with reduced discharge variability after a stroke (33, 34). These changes directly affect the electrical activity and might lead to reduced muscle force and disability in subtle responses to the perturbations during functional tasks. Here, the reduction in fApEn values was reflected in the decreased complexity of the EMG signals of poststroke subjects, which might be related to the alternations in the properties of motor units. Our findings were consistent with those reported by Ao et al., who found lower fApEn values in elbow muscles of poststroke subjects compared to healthy controls during trajectory-tracking tasks. This could be attributed to a reduced number and firing rate of active motor units (29). Similarly, the decreased complexity of EMG signals was also reported in other patients with neuromuscular disorders, such as Parkinson’s disease (35) and cerebral palsy (36), which could be explained by the disease-induced muscle fiber degeneration (37).

During swing phase, the fApEn values for the BF, RF, and TA (20 and 30% leg length) in the leading limb were higher in poststroke subjects than in healthy controls. This might be as a result of abnormal gait during obstacle crossing after a stroke. The swing phase of the leading limb during obstacle crossing caused the subjects to elevate their lower limb to secure sufficient toe-obstacle clearance, and this could be a challenge for poststroke subjects, leading to abnormal muscle activation patterns. Indeed, our previous study found that to avoid falling, toe-obstacle clearance of stroke survivors was greater than in healthy controls (38). The increased activation of thigh muscles in the BF and RF was found in stroke survivors (14). This might also contribute to the increased complexity of the EMG signals (36).

### fApEn Values Change with Task Difficulties

When sustaining different levels of maximal voluntary contraction force in the upper limb, the complexity of EMG signals had been demonstrated to increase with increasing muscle contraction forces (23, 37, 39). In line with these findings, our results demonstrated that increasing obstacle heights demanded an increase in muscle contraction forces that in turn led to the recruitment of more motor units and increased firing rates in active motor units (14). Our results suggested that the complexity of the EMG signals increased with greater task demands, and this could also be applied to poststroke subjects. Therefore, safely crossing higher height obstacles requires increased muscle contraction forces and more activated motor units, leading to higher entropy values for the EMG signals (37).

However, there are still discrepancies about the complexity changes with task difficulties. To investigate the effect of task demands on motor entropy, Hong and Newell found that the entropy values of muscle forces decreased as the task demands increased (40). They explained the decreased entropy with increased task demands, but reduced environmental information, revealing a compensatory interaction between tasks and the environment on the force dynamics. Moreover, Murillo et al. found that fuzzy entropy of postural sway in healthy young adults decreased from the stable condition to the mid-level instability condition. This increased again at the highest instability condition at the anterior–posterior axis, which reflects the adaptations of postural control system to the platform instability (41). Therefore, the compensatory and adaptive nature of the motor control system to the task complexity warrants further investigation, especially in stroke survivors. Entropy analysis could be used to evaluate the effects of rehabilitation interventions targeting the motor recovery to restore complex motor tasks in persons after a stroke.

### Limitations

There were several limitations in this study. First, considering the insufficient strength of the paretic leg during the stance phase, we did not instruct the poststroke subjects to first step over the obstacle with their unaffected side due to safety issue. Thus, we could not compare the paretic side with the unaffected side during the same task. In the future, we should introduce stroke subjects to first step over the obstacle with both affected and unaffected limbs. Second, moderate to high functional level of persons after stroke were recruited in this study. A large-scale study of different types of stroke subjects should be recruited in future study to investigate the influences of group and obstacle height, which may help explore the mechanisms and guide rehabilitation after stroke.

## Conclusion

In this study, the stoke-related changes in complexity of lower muscles during obstacle crossing were investigated using fApEn. Results show that the complexity of RF in trailing limb during stance phase decreased in stroke group, which might be associated with the reduced number and firing rate of MU. However, during the swing phase, there were non-significant increases in the fApEn values of BF and RF in the trailing limb of the stroke group, resulting in a coping strategy when facing challenging tasks. During the gait, the complexity of muscle activation increases with obstacle height. That might be because higher obstacles demand greater muscle forces, which causes more motor units to be recruited and triggers higher firing rates of motor units. These findings based on the fApEn values of the EMG signals indicate that the complexity analysis using fApEn could be a suitable and non-invasive method to evaluate muscle function changes after stroke.

## Ethics Statement

The study was approved by the Ethics Committee of the First Affiliated Hospital of Sun Yat-sen University. The study was conducted in accordance to the Declaration of Helsinki. All subjects provided written informed consent prior to enrollment.

## Author Contributions

YC, CM, and NC conceived and designed the study and performed the experiments. YC and HH wrote the paper. RS and YZ contributed to experiments. RS and LL reviewed and edited the manuscript. All authors had read and approved the manuscript.

## Conflict of Interest Statement

All financial, commercial, or other relationships that might be perceived by the academic community as representing a potential conflict of interest must be disclosed. If no such relationship exists, authors will be asked to confirm the following statement.
